# Sexual dysfunction and anxiety in HIV-1-infected males in Eastern Sicily

**DOI:** 10.1186/1758-2652-13-S4-P109

**Published:** 2010-11-08

**Authors:** BM Celesia, C Coco, F Bisicchia, G Pellicanò, MT Mughini, F Palermo, G Nunnari, R Russo

**Affiliations:** 1 Unit of Infectious diseases University of Catania ARNAS Garibaldi, via Palermo 636, Catania, Italy; 2 A.O.U. 'G. Martino' Policlinico, Unit of Infectious diseases, University of Messina, Messina, Italy

## Purpose of the study

Sexual dysfunctions (SD) in HIV-1 infected individuals have been associated with infection, modification of body image and HAART, mainly PI based treatment. Anxiety is common in HIV+ subjects.

We hypothesized that anxiety could be a cofactor of SDs. A cross sectional analysis was conducted in a cohort of 109 HIV+ males with at least 6 months of continuous exposure to HAART to evaluate the prevalence of anxiety and other factors associated with SDs.

## Methods

Sexual evaluation was performed by self-completion questionnaires International Index of Erectile Function (IIEF): a score of <26 was considered diagnostic of erectile dysfunction (ED). Anxiety was evaluated with Self Rating Anxiety State SAS 054, a self-submitted 20 items questionnaire. A z score 45 was considered diagnostic of anxiety.

## Results

109 male patients were enrolled. Median age 46 (IQR 40-52) years, 29% of 50 years old, 25% heterosexuals, 54% MSMs, 22% IVDUs; 45% single. Median time from HIV diagnosis was 11 years (IQR 5-15). 50% CDC A, 20% CDC B, 30% CDC C. Median CD4 cell count 577 (IQR 383-861) cells/µl, 80% had HIV RNA <50 copies/ml. Median length of HAART was 8 years (IQR 4-13): 22% were naïve, 23% on 2nd line, 28% 3rd-4rd line, 27% >4th line of treatment. 61% were on PI based treatment, 38% on NNRTI. Permanence on PI was 74 months (IQR 40-124), on NNRTI 47 months (IQR 18-87). 52 subjects (48%) had a z score of 45 diagnostic of anxiety.

71 subjects (65%) had ED. EDs were more frequent in elderly subjects (>50 yr) (78% vs 60%, OR 2.7, 95% CI 1.01-2.26) and in individuals with anxiety disorders (77% vs 54%, OR 3.04, 95% CI 1.3-7.12). No significant association was seen with actual ARV treatment, length of time on PI or NNRTI, clinical stage, HIV RNA >50 copies/ml.

## Conclusions

EDs were highly prevalent and related to elderly age (P<0.05) and anxiety (P<0.01). We do not show any correlation with actual ARV treatment and with length of exposition time to PI or NNRTI based treatment.

An accurate evaluation and treatment of anxiety should be considered and offered in order to obtain an increase in sexual satisfaction and in quality of life of HIV-1-infected patients.

